# Mechanistic Insights Behind the Self-Assembly of Human Insulin under the Influence of Surface-Engineered Gold Nanoparticles

**DOI:** 10.1021/acschemneuro.4c00226

**Published:** 2024-05-10

**Authors:** Zachary Flint, Haylee Grannemann, Kristos Baffour, Neelima Koti, Emma Taylor, Ethan Grier, Carissa Sutton, David Johnson, Prasad Dandawate, Rishi Patel, Santimukul Santra, Tuhina Banerjee

**Affiliations:** † Department of Chemistry and Biochemistry, Missouri State University, 901 S. National Avenue, Springfield, Missouri 65897, United States; ‡ Molecular Graphics and Modeling Laboratory, University of Kansas, 2034 Becker Drive, Lawrence, Kansas 66018, United States; § Department of Cancer Biology, The University of Kansas Medical Center, Kansas City, Kansas 66160, United States; ∥ Jordan Valley Innovation Center, Missouri State University, 542 N. Boonville Avenue, Springfield, Missouri 65806, United States

**Keywords:** insulin fibrillation, amyloid protein, bionano interface, molecular dynamics simulations, neurodegenerative disorders

## Abstract

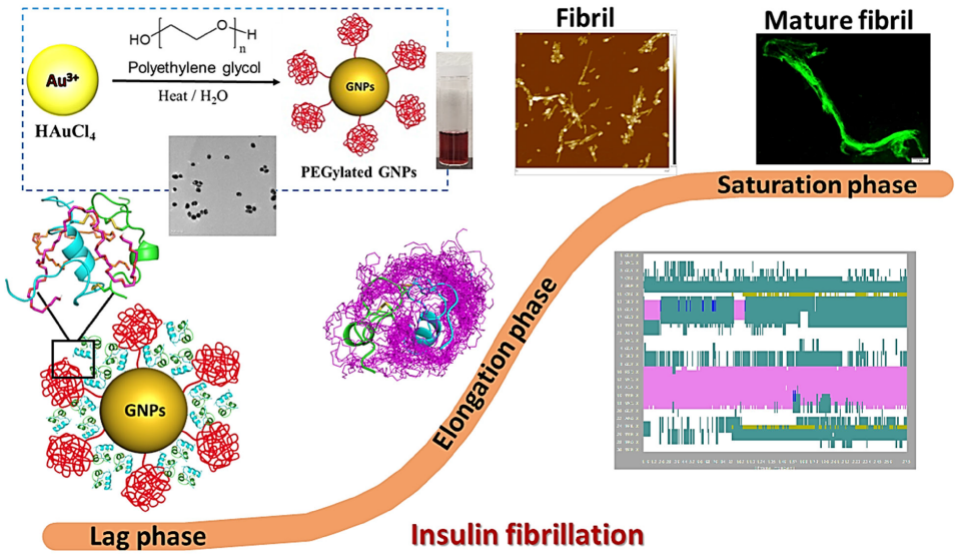

Elucidating the underlying principles of amyloid protein self-assembly at nanobio interfaces is extremely challenging due to the diversity in physicochemical properties of nanomaterials and their physical interactions with biological systems. It is, therefore, important to develop nanoscale materials with dynamic features and heterogeneities. In this work, through engineering of hierarchical polyethylene glycol (PEG) structures on gold nanoparticle (GNP) surfaces, tailored nanomaterials with different surface properties and conformations (GNPs-PEG) are created for modulating the self-assembly of a widely studied protein, insulin, under amyloidogenic conditions. Important biophysical studies including thioflavin T (ThT) binding, circular dichroism (CD), surface plasmon resonance (SPR), and atomic force microscopy (AFM) showed that higher-molecular weight GNPs-PEG triggered the formation of amyloid fibrils by promoting adsorption of proteins at nanoparticle surfaces and favoring primary nucleation rate. Moreover, the modulation of fibrillation kinetics reduces the overall toxicity of insulin oligomers and fibrils. In addition, the interaction between the PEG polymer and amyloidogenic insulin examined using MD simulations revealed major changes in the secondary structural elements of the B chain of insulin. The experimental findings provide molecular-level descriptions of how the PEGylated nanoparticle surface modulates protein adsorption and drives the self-assembly of insulin. This facile approach provides a new avenue for systematically altering the binding affinities on nanoscale surfaces by tailoring their topologies for examining adsorption-induced fibrillogenesis phenomena of amyloid proteins. Together, this study suggests the role of nanobio interfaces during surface-induced heterogeneous nucleation as a primary target for designing therapeutic interventions for amyloid-related neurodegenerative disorders.

## Introduction

Self-assembly of proteins and peptides is a ubiquitous phenomenon and occurs in several biological processes.^1−4^ Amyloid formation, a special category in the self-assembly process, is known to be modulated in the presence of surrounding interfaces such as cell membranes, vesicles, microparticles, and nanostructured surfaces. Understanding the mechanisms of amyloid formation at nanobio interfaces remains extremely challenging due to the interplay of several dynamically interacting components. Important aspects that are critical for characterizing such interface events include intrinsic physicochemical properties of nanomaterials and their dynamic interactions that gives nanoscale objects a separate entity when they encounter biological systems.^5,6^

Though the process of self-assembly of amyloidogenic proteins has been extensively investigated, yet further insights on the origin of the remarkable thermodynamic stability associated with the amyloid fibrils is necessary for designing better therapeutics.^7,8^ The spontaneous growth of amyloid protein aggregates has proven an elusive target within the field of antiamyloid drug development. Since the implication of this virtually irreversible assembly of native proteins in a variety of neurodegenerative disorders, research has sought to illuminate the structural motifs and mechanism of formation exhibited by these aggregates.^9−14^ Structural determinations have yielded models of both amorphous aggregates and fibrillar structures. Amorphous aggregates and amyloid fibrils are two major categories of aberrant aggregates that are associated with several neurodegenerative diseases including Alzheimer’s disease and Parkinson’s disease.^15,16^ In both cases, individual monomers adopt a non-native, β-sheet-rich conformation that facilitates intermolecular interactions that are integral to the lattice stability. This β-sheet conformation is highly unfavorable for free monomers, which has prompted many inquiries concerning the mechanistic steps that allow for this transition.^17,18^ The current consensus involves a stochastic nucleation event incited by the collision of transient, partially folded monomers, which arises due to the disordered nature of the proteins typically observed to have higher propensities for amyloid aggregation. These collisions inevitably affect metastable oligomers, which serve as a platform for aggregation. In addition, some have postulated that the binding mode exhibited by the oligomer likely determines how the aggregation proliferates.^19−21^

Insulin is only of such proteins with a tendency for amyloid fibril formation, which take the form of waxy deposits on pancreatic tissue in patients with type II diabetes.^22^ Additionally, precipitation of insulin aggregates at injection sites has been reported in some patients.^23−26^ These deposits serve to exacerbate symptoms and mitigate the need for treatment. Furthermore, recent evidence suggests that insulin fibrils can potentially seed the formation of α-synuclein, which could reveal a link between diabetes and Parkinson’s disease.^27^ As problematic as all that is, strategies for reducing the rate of insulin fibril formation are in high demand. Because of this, studies aimed at delineating the kinetic factors responsible for nucleation and subsequent growth have become valuable in recent years. Emerging nanotechnologies, such as metal nanoparticles and quantum dots, have provided means of introducing amyloid proteins into novel environments.^28,29^ Ever since the pioneering work of Linse,^30^ where it was shown that nanoparticles can act as conventional catalysts for protein fibrillation, several studies have demonstrated nanoscale materials can significantly modulate the nucleation and aggregation mechanisms of amyloid proteins and peptides and exhibit different kinetic effects including retardation and acceleration depending on the physicochemical characteristics of the nanomaterials and properties of proteins in solution.^31−37^ Specifically, reports on functionalized gold nanoparticles (GNPs) have demonstrated the ability to modulate fibril formation. PEGylated nanoparticles have also become a potential candidate for modulation as their biocompatibility has been thoroughly demonstrated by a variety of drug delivery systems.^38−40^ Although the effect of different surfaces and nanoparticles with varied chemical composition and sizes on amyloid formation kinetics have been studied, the impact of nanoparticle’s topographical structures on fibrillation properties of amyloidogenic peptides and proteins remains elusive.

Herein, we introduce a modular approach for studying the effect of distinct surface features on self-assembly mechanisms. Specifically, the implications of GNP surface charge and PEG conformations with dynamic features on the self-assembly of amyloidogenic insulin are examined using a combination of experimental and computational modeling studies. Insulin is being examined due to its ability to readily form amyloid fibrils in kinetic assays under certain experimental conditions^37^ (low pH and elevated temperature), as well as its pathogenic relevance in type II diabetes. Amyloid assembly kinetics, secondary structural changes, and morphology of the resultant fibrils were investigated using biophysical and microscopy techniques. Molecular modeling studies were performed for the assessment of conformational dynamics of proteins in the presence of PEG-tunable nanostructures and elucidation of interactions. Topological conformations engineered on the surface of the nanoparticles are important aspects of the design of this study. This can be further modulated by altering the core properties and surface density for diverse applications including the investigation of interfacial interactions of other amyloid proteins and functional amyloids and for tuning fibrillation properties in diverse biological systems.

## Results and Discussion

### PEG Tunable Nanostructures and Their Possible Interaction Mechanism with Insulin

**Scheme 1:**
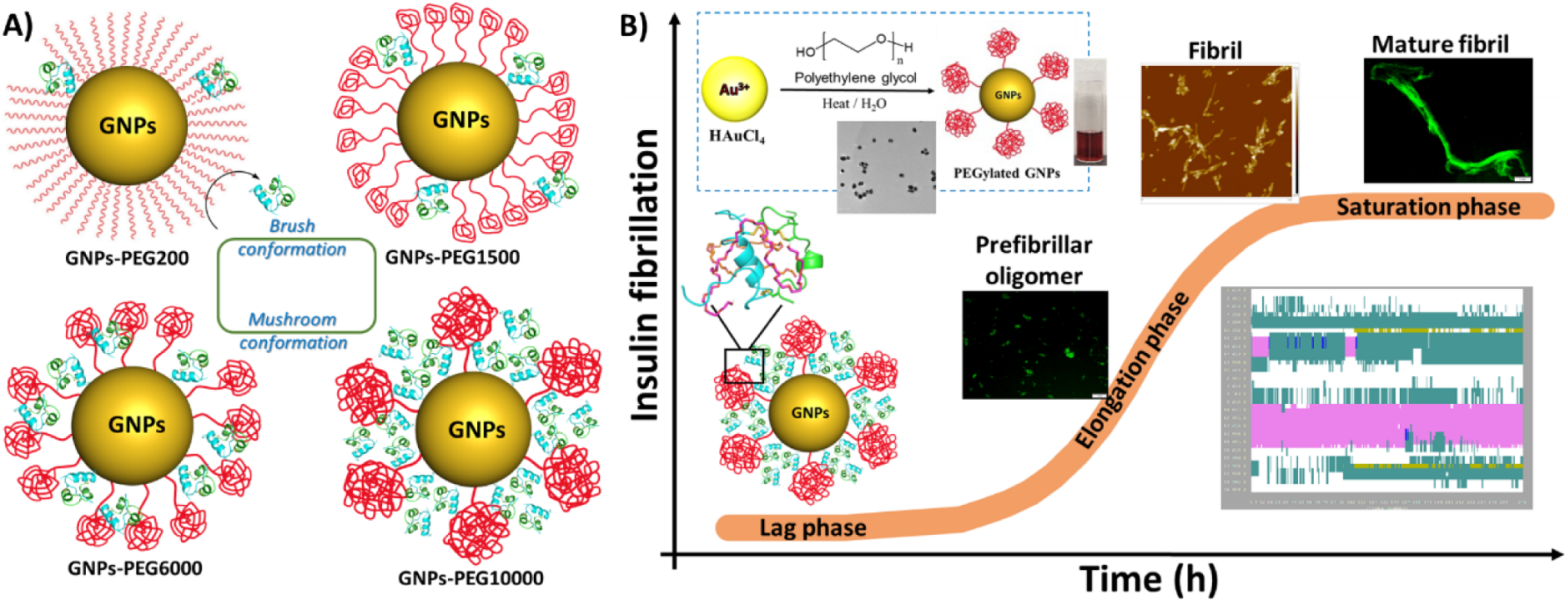
(**A**) Schematic Presentation of the Proposed *Brush* and *Mushroom* Conformations of GNPs-PEG and Possible Interactions with Insulin. (**B**) GNPs-PEG Synthesis (inset) and Nanoscale Interaction Kinetics between Insulin and GNPs-PEG

The amyloid folding state represents a highly unfavorable configuration for free monomers of amyloidogenic proteins. Therefore, a substantial energy barrier exists between native monomers and amyloid aggregates.^7,8^ This barrier forms the basis for the observed “lag phase” in amyloid fibrillation as the first step of the reaction requires random folding of monomers in an uphill direction with respect to the free energy of the system. As these misfolded monomers associate into dimers, trimers, and higher-order oligomers, a critical concentration of amyloid-state proteins is approached, when autocatalysis takes over and the fibrils undergo rapid growth (referred to as “elongation”).^29−32^ Self-assembly of insulin protein into amyloid fibrils under the influence of PEG tunable dynamic nanostructures can be represented with a sigmoidal growth curve, as shown in Scheme 1.

To investigate the impact of dynamic nanobio interfaces on insulin adsorption and nucleation, hierarchical PEG conformations (*Brush* and *Mushroom*) on the surface of the gold nanoparticles are introduced by altering the molecular weight of the PEG polymer from low to high, ∼200–10000 Da (Scheme 1A). We hypothesized that the densely grafted PEG chains constructed using low molecular weight PEG result in “*Brush*” conformations and, therefore, prevent adsorption of proteins on the nanoparticle surface due to higher configurational entropy of the flexible polymer chains. On the other hand, in the “*Mushroom*” regime, the higher molecular weight PEG chains adopt coiled conformations and hence facilitate higher adsorption of amyloid proteins on the nanoparticle surface due to availability of void space in between the coiled PEG chains.^37,40^ It is further hypothesized that such engineering at nanoscale surfaces will best offer the possibility of achieving differential kinetic effects during the amyloid formation of insulin protein. Specifically, we present the novel molecular mechanisms of insulin self-assembly into amyloid fibrils in the presence of GNPs-PEG. The combined effect of surface charge and PEG chain length on the fibrillogenisis pathway of insulin was examined using a combination of spectroscopy, microscopic, and molecular modeling studies (Scheme 1B).

### Synthesis and Characterizations of GNPs-PEG

An efficient one-step synthetic protocol was adopted to synthesize GNPs-PEG, as shown in Scheme 1. Unmodified PEG polymer of different chain length (200–10000 Da) was used as a reducing agent as well as a stabilizing agent to tune the surface charge without any alteration in the colloidal stability.^27,28^ Morphology, average particle size, zeta potential, and absorption maximum of synthesized polymer-coated nanoparticles were characterized by DLS, TEM, and UV–vis spectroscopy (Figure S1). Representative TEM image (Figure S2) showed spherical morphology, and DLS studies indicated that all the GNPs-PEG preparations were approximately of similar size distribution. Moreover, all the synthesized GNPs-PEG possess negative zeta potential due to the presence of −OH groups on the surface and neighboring water molecules at the slipping planes. As summarized in Table S1, GNPs-PEG_200_ was found to have a high negative surface charge of −25.2 mV, and with the increase in the PEG chain length, this value became less negative (GNPs-PEG_10000_: −5.1 mV). This is due to higher chain lengths and functional group ratio. UV–vis spectra (localized surface plasmon resonance: LSPR) of all PEG-coated GNPs exhibited a well-defined absorption band between λ_abs_: 520–524 nm. Colloidal gold nanoparticles exhibit distinct optical properties that are induced by localized surface plasmon resonance (LSPR). LSPR occurs due to the collective oscillation of conduction electrons on the surface of gold nanoparticles when irradiated with visible light. LSPR peak (absorption maximum) of synthesized GNPs-PEG was determined through UV–vis measurements. Higher change in LSPR for short-chain PEG (PEG_200_) further indicates that due to higher grafting density, it can induce more changes in the dielectric constant of the medium surrounding the surface of GNPs. The stability of the GNPs-PEG was assessed by visual dispersion and measuring change in diameters after 60 days at pH 7.4 and 3.0, respectively, and the results are summarized in Table S2.

### DLS Analysis of Insulin Fibrillation in the Presence of GNPs-PEG

**Figure 1:**
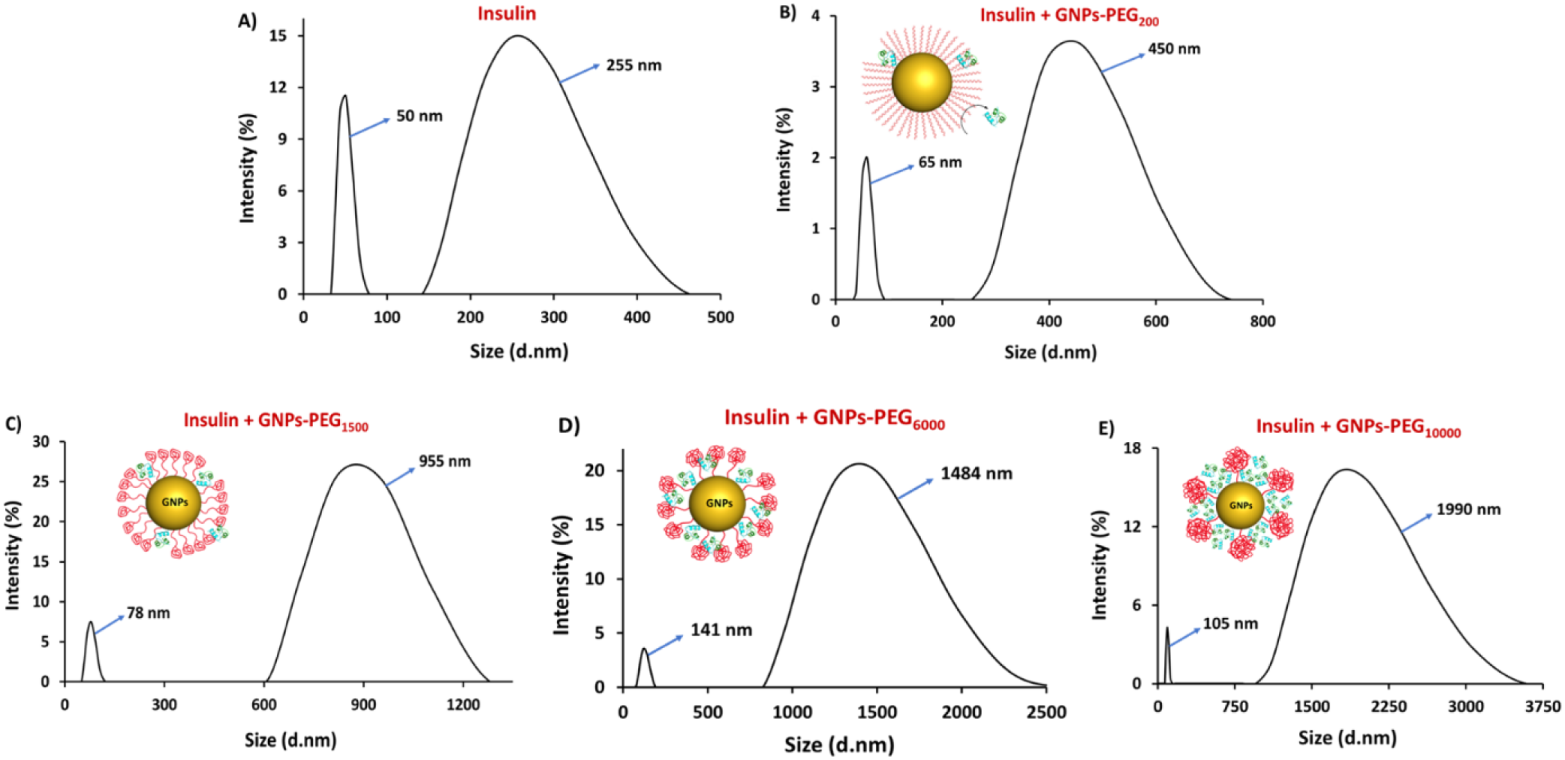
Representative DLS profiles of insulin during fibrillation in the (**A**) absence and (**B**, **C**, **D**, and **E**) presence of GNPs-PEG.

The average size distribution of insulin aggregates in the presence of GNPs-PEG was determined by using DLS measurements. As shown in Figure 1, two well-defined aggregate peaks were observed for insulin alone after 3 h of incubation at 50 °C (Figure 1A). Coincubation of insulin with the GNPs-PEG was seen to induce fibrillation at a higher rate as bigger size aggregates were observed. GNPs-PEG_10000_ resulted in the bigger size fibril aggregates (d_H_ of 105 and 1990 nm) and was significantly larger than GNPs-PEG_200_. Size distribution pattern of insulin with and without GNPs-PEG showed peaks between 50 and 141 nm, suggesting that fibrillogenisis possibly proceeds through the formation of oligomeric intermediates.

### Thioflavin T Assay: Insulin Fibrillation

**Figure 2:**
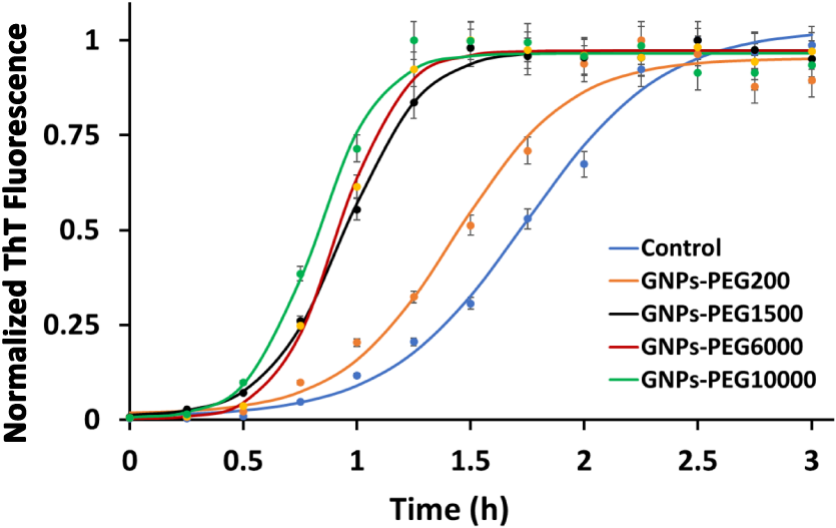
Representative ThT kinetic profiles of insulin fibrillation in the absence and presence of 0.2 nM GNPs-PEG. The graph illustrates mean ± SD (*n* = 3 replicates). ThT experiments in the presence and absence of nanoparticles were repeated three times using different batches of GNPs-PEG with three replicates at each time point.

Self-assembly behavior of human insulin under amyloidogenic conditions was determined in the absence and presence of GNPs-PEG using thioflavin T (ThT) binding assays. ThT is an amyloid-specific fluorescent dye that allows for real-time monitoring of fibrillation.^41−44^ It is known to fluoresce weakly in its free state; however, upon binding with amyloid fibrils, there is significant enhancement in its fluorescence intensity. The ThT fluorescence emission of insulin fibrillation was collected in the absence (control) and presence of GNPs-PEG over a period of 3 h at 50 °C by exciting at λ_ex_ = 450 nm (Figure 2). The kinetic data showed the growth of insulin fibrils proceeded via a nucleation-dependent step in the absence of GNPs-PEG, where at the beginning until 1 h, no significant change in the ThT fluorescence was observed. This phase was referred to “lag time,” which is then followed by a growth phase commonly known as “elongation time.” After the growth phase, the ThT fluorescence reached a saturation phase. By fitting the ThT fluorescence data to sigmoidal equation, the lag time was found to be 1.13 ± 0.10 h in the absence of GNPs-PEG (Table 1). However, in the presence of GNPs-PEG, insulin showed faster fibrillation, and lag times were found to be shorter, 0.61 ± 0.05 h and 0.54 ± 0.09 h for GNPs-PEG_6000_ and GNPs-PEG_10000_, respectively. It is evident that fibrillation of insulin in the presence of GNPs-PEG proceeds via the formation of oligomeric species in a shorter lag phase. Interestingly, the slope of the growth phase for higher chain length PEGs (6000, 10000 M.W) was found to be less steep due to *Mushroom* conformation-facilitated higher adsorption of insulin monomers, which in turn leads to faster nucleation.

**Table 1:** Kinetics of Insulin Fibrillation in the Absence (Control) and Presence of PEGylated GNPs Suggest Nanoparticles Accelerate Fibril Formation by Shortening the Lag Phase^a^

experiment	control	**GNPs-PEG**_**200**_	**GNPs-PEG**_**1500**_	**GNPs-PEG**_**6000**_	**GNPs-PEG**_**10000**_
*T*_1/2_ (h)	1.74	1.43	0.94	0.91	0.82
lag time (h)	1.13 ± 0.10	0.93 ± 0.10	0.65 ± 0.10	0.61 ± 0.05	0.54 ± 0.09

a The error bars represent standard deviation from three replicates that were performed.

### Assessment of Insulin Fibrillation by Congo Red Binding

**Figure 3:**
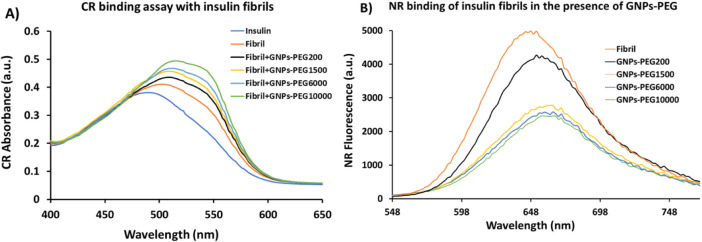
(**A**) Congo red absorption and (**B**) Nile red fluorescence of insulin fibrillations in the presence and absence of GNPs-PEG. Results acquired in these experiments are in triplicate and represent the mean ± standard deviation.

Congo red (CR) binding assay^45,46^ was used to evaluate the extent of aggregation induced in the presence of GNPs-PEG. As shown in Figure 3A, the absorption peak of CR in insulin at 0 h (insulin sample incubated with CR at 50 °C and pH 3.0) was at 490 nm, suggesting the absence of fibrillar aggregates. It was observed that this absorption maximum red shifted about 14 nm when incubated for 3 h. Moreover, the red shift became more prominent when insulin was incubated with GNPs-PEG at 50 °C and pH 3.0 for 3 h and showed noticeable increase in the absorbance, indicating increase in the β-sheet structure in the presence of these nanoparticles. Broader peaks were obtained in the presence of GNPs-PEG, which was attributed to the presence of mixtures of fibril-bound and fibril-free CR molecules.

### Nile Red Fluorescence Assay for Insulin Fibrillation

To investigate the surface exposure of hydrophobic residues during insulin fibrillation in the presence of GNPs-PEG, Nile red (NR) fluorescence assay was performed.^47^ NR, a sensitive fluorescent dye, is known to show several fold enhancements in fluorescence intensity upon binding to the exposed hydrophobic surface of the protein along with a blue shift. NR fluorescence emission data (Figure 3B) showed a decrease in the intensity with GNPs-PEG_10000_ compared with the control. These results suggested that the solvent-exposed hydrophobic surface of insulin oligomers and fibrils decreases in the presence of GNPs-PEG nanoparticles.

### CD Spectroscopy of Insulin in the Presence of PEGylated GNPs

**Figure 4:**
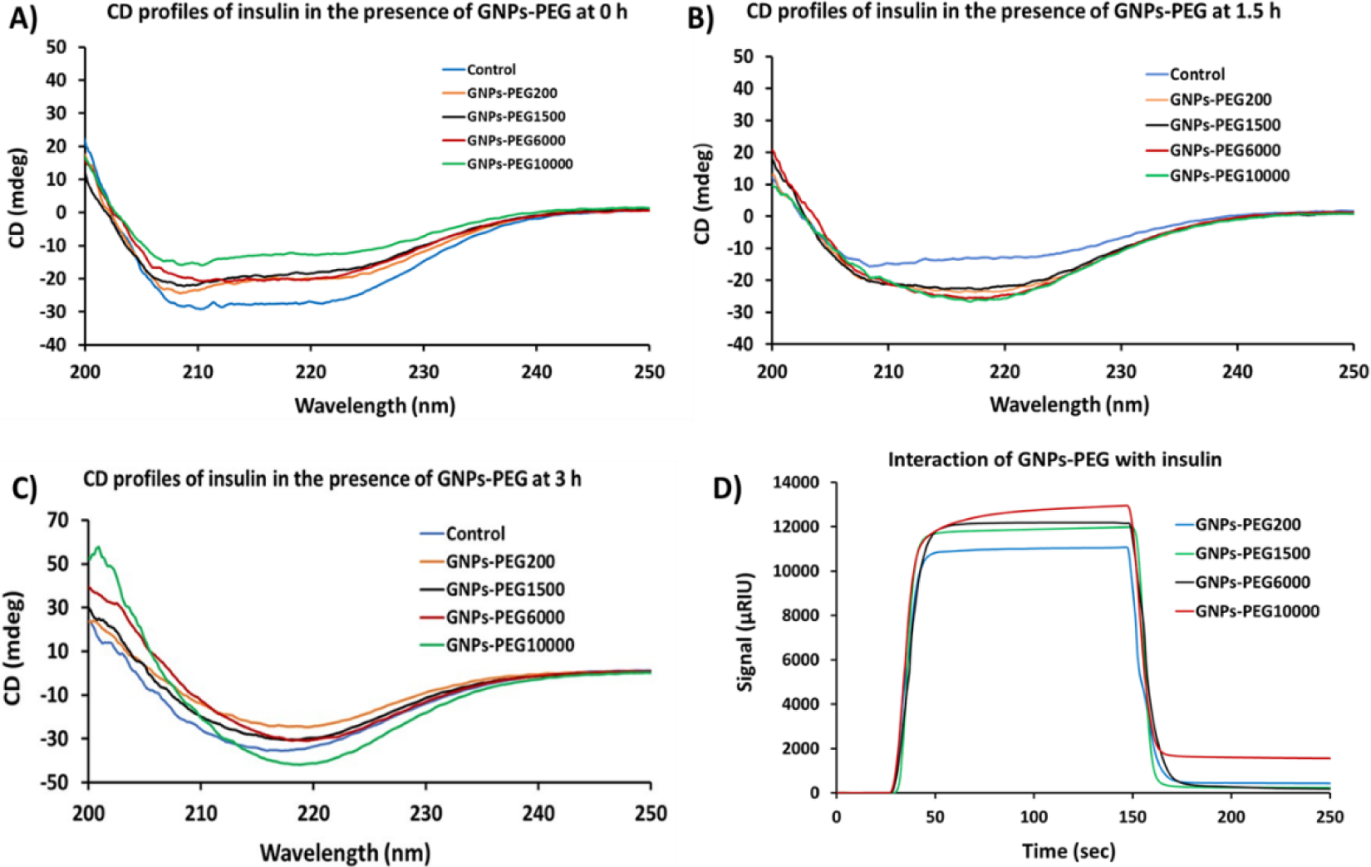
Representative CD spectra of insulin without (control) and with PEGylated GNPs at (**A**) 0 h, (**B**) 1.5 h, and (**C**) 3 h. (**D**) Representative SPR sensorgrams showing interactions between insulin and GNPs-PEG. Binding experiments were repeated in triplicates.

The effect of the nanobio interface of GNPs-PEG on the secondary structure of insulin was investigated using far-UV CD spectra. As shown in Figure 4A, insulin incubated at 0 h (50 °C, pH 3.0) without (control) and with GNPs-PEG had characteristic far-UV spectra of a typical α-helical protein with two characteristic minima at 208 and 222 nm. Significant α-helix to β-sheet transition was observed after 1.5 h of incubation, and it was more pronounced in the presence of higher molecular weight PEG-coated GNPs. Interestingly, the helical conformation, as indicated by the presence of negative band at 208 nm, was observed for insulin only and insulin incubated with GNPs-PEG_200_ after 1.5 h. However, more β-rich intermediates were observed for higher molecular weight PEGylated GNPs (Figure 4B), suggesting GNPs-PEG promote the collapse of the α-helical structure. Furthermore, CD spectroscopy results indicated that after 3 h of incubation, there was a rapid conversion of the secondary structure of insulin into the β-rich fibrillar aggregates (Figure 4C). There are two barriers during insulin fibrillation: the first being the collapse of the α-helix structure and the second being the formation of the β-sheet structure. To examine whether these nanostructures can solely promote β-sheet structure formation, future studies are underway on GNPs-PEG interactions with intrinsically disordered amyloid proteins and their role in the conformational properties.

### Surface Plasmon Resonance Experiments

To examine the mechanistic origin of the combined effect of surface charge and PEG conformations on the formation of amyloid fibrils from human insulin, SPR studies were performed. Insulin monomers were immobilized on CM5 sensor chip, and GNPs-PEG were then injected. The sensorgram of each GNPs-PEG showed a pattern of association and dissociation. The response curve of GNPs-PEG_10000_ showed highest increase, whereas GNPs-PEG_200_ displayed lowest response, which indicates weaker binding with GNPs-PEG_200_ and strong binding with GNPs-PEG_10000_ (Figure 4D). Difference in the binding response was attributed to the molecular size and conformation of PEG chain^48^ bound to GNP surface that eventually dictates its interaction with insulin monomers. Overall, low molecular weight PEG is expected to result in densely packed PEG chains with *Brush* conformations that ultimately result in less binding/adsorption of insulin monomers.

### Molecular Modeling Experiments: MD Simulations

**Figure 5:**
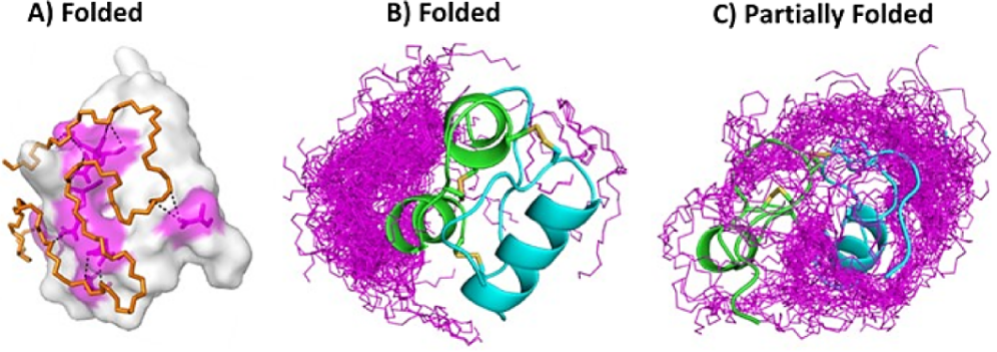
Potential interactions of PEG polymers with insulin. (**A**) One model of the interaction between PEG and properly folded insulin, highlighting how PEG wraps can make many surface contacts with insulin as well as hydrogen bonds (dashed lines). (**B**) The top 100 scoring poses of PEG to properly fold insulin from 1000 independent docking simulations. (**C**) The top 100 scoring poses of PEG to partially folded insulin (amyloidogenic conditions) from 1,000 independent docking simulations.

The interaction between linear segments of PEG and insulin was further explored using molecular docking with Autodock, followed by molecular dynamics (MD) simulations. To explore any potential interaction, PEG1500 molecules were docked to a crystallographically solved structure of insulin^49^ PDB 3I40 using Autodock Vina,^50^ enabling PEG1500 to sample the entire surface of insulin. Figure 5A shows an example conformation selected due to its score and a larger number of hydrogen bonds.

Because of the size of PEG1500 and its flexibility, the potential interaction between insulin and linear PEG chains was explored much more thoroughly by performing 1000 docking simulations, with more thorough sampling in each run, both a crystallographically solved structure of insulin, PDB 2G4M,^51^ as well as one member of the NMR ensemble of human insulin under amyloidogenic conditions, PDB 1SF1.^52^ To reduce the computation time of docking and subsequent MD analysis, shorter simulations for each insulin conformation were pooled, and the top scoring poses were assessed for a consensus among the best scoring poses. The top scoring poses did not converge to a consensus solution; instead, they clustered around regions on the protein surface, suggesting a nonspecific interaction. That said, there was a stark difference in the interaction patterns with properly folded insulin versus those with amyloidogenic insulin. Linear PEG interacted almost exclusively with the A chain of insulin, while it interacted more with the B chain at the interface of chains A and B (Figure 5B,C). When interacting with properly folded insulin, the top 100 scoring PEG chains were modeled to predominantly wrap around Tyr14 of chain A. However, when interacting with partially folded (amyloidogenic) insulin, PEG was able to interact much more with the newly exposed hydrophobic surfaces.

**Figure 6:**
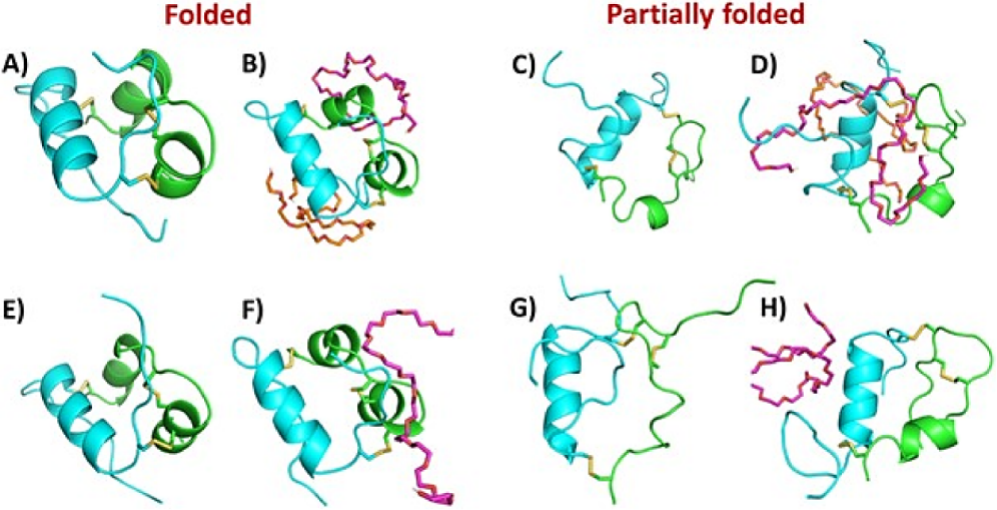
Initial and final conformations of the 25 ns molecular dynamics (MD) simulations. Initial conformations of (**A**) folded insulin without PEG, (**B**) folded insulin with 2 PEG molecules, (**C**) partially folded insulin without PEG, and (**D**) partially folded insulin with two PEG molecules and final conformations of (**E**) folded insulin without PEG, (**F**) folded insulin with 2 PEG molecules, (**G**) partially folded insulin without PEG, and (**H**) partially folded insulin with two PEG molecules are shown. Chain A is represented as a green cartoon, and chain B is represented as a cyan cartoon. Disulfide bonds are shown as sticks. PEG molecules are shown as magenta and orange sticks.

**Figure 7:**
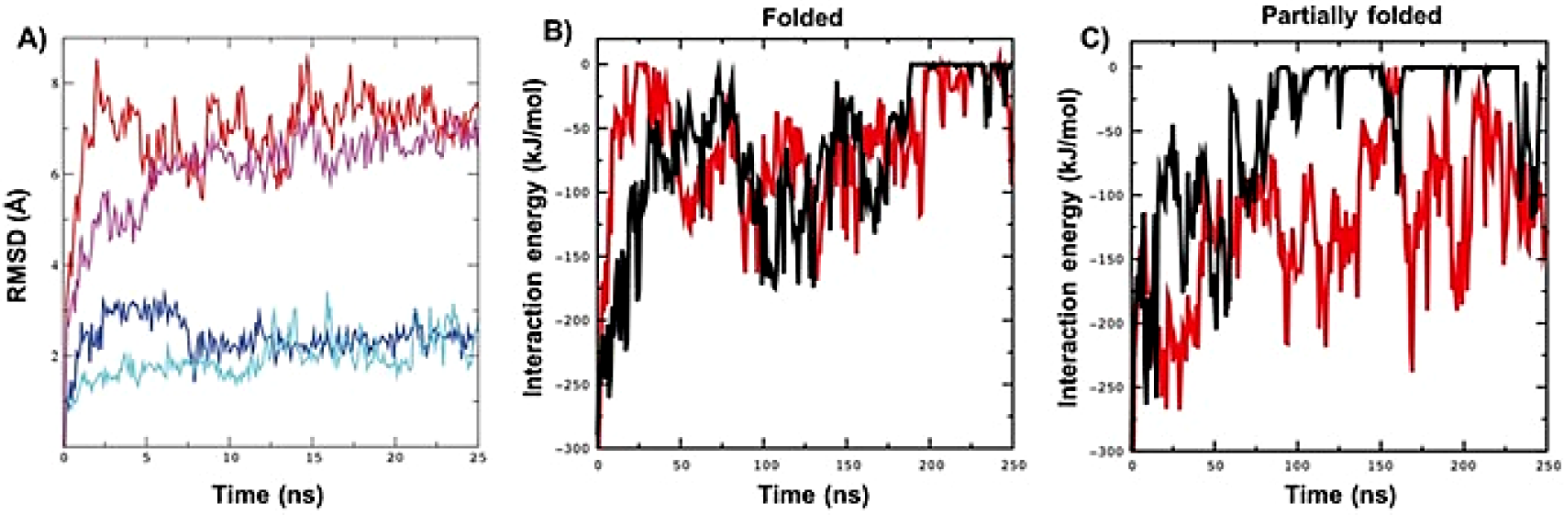
RMSD and the interaction energy over time. (**A**) RMSD from starting structures over time. The RMSD (Å) from the initial structures are plotted over time for the 4 molecular dynamics (MD) simulations: (blue) folded insulin without PEG, (cyan) folded insulin with 2 PEG molecules, (red) partially folded insulin without PEG, and (magenta) partially folded insulin with two PEG molecules. (**B–C**) Approximations of the interaction energies between insulin and individual PEG molecules over time. The sum of short-range Lennard–Jones and Coulombic potentials are plotted over time between individual PEG molecules (blue and black) and (**B**) folded insulin and (**C**) partially folded insulin.

Molecular dynamics was used to further probe these potential interactions. Two nonoverlapping PEG molecules were selected from each docked system, and a 25 ns simulation was performed to explore the interactions and effects of linear PEG molecules on properly folded and partially folded insulin. Twenty-five ns simulations were also performed in the absence of PEG to use as a baseline for comparison. The backbone of properly folded insulin changes relatively little with and without PEG, while partially folded insulin was noticeably different (Figure 6), as reflected in the tripling in RMSD values between folded and partially folded insulin (Figure 7).

**Figure 8:**
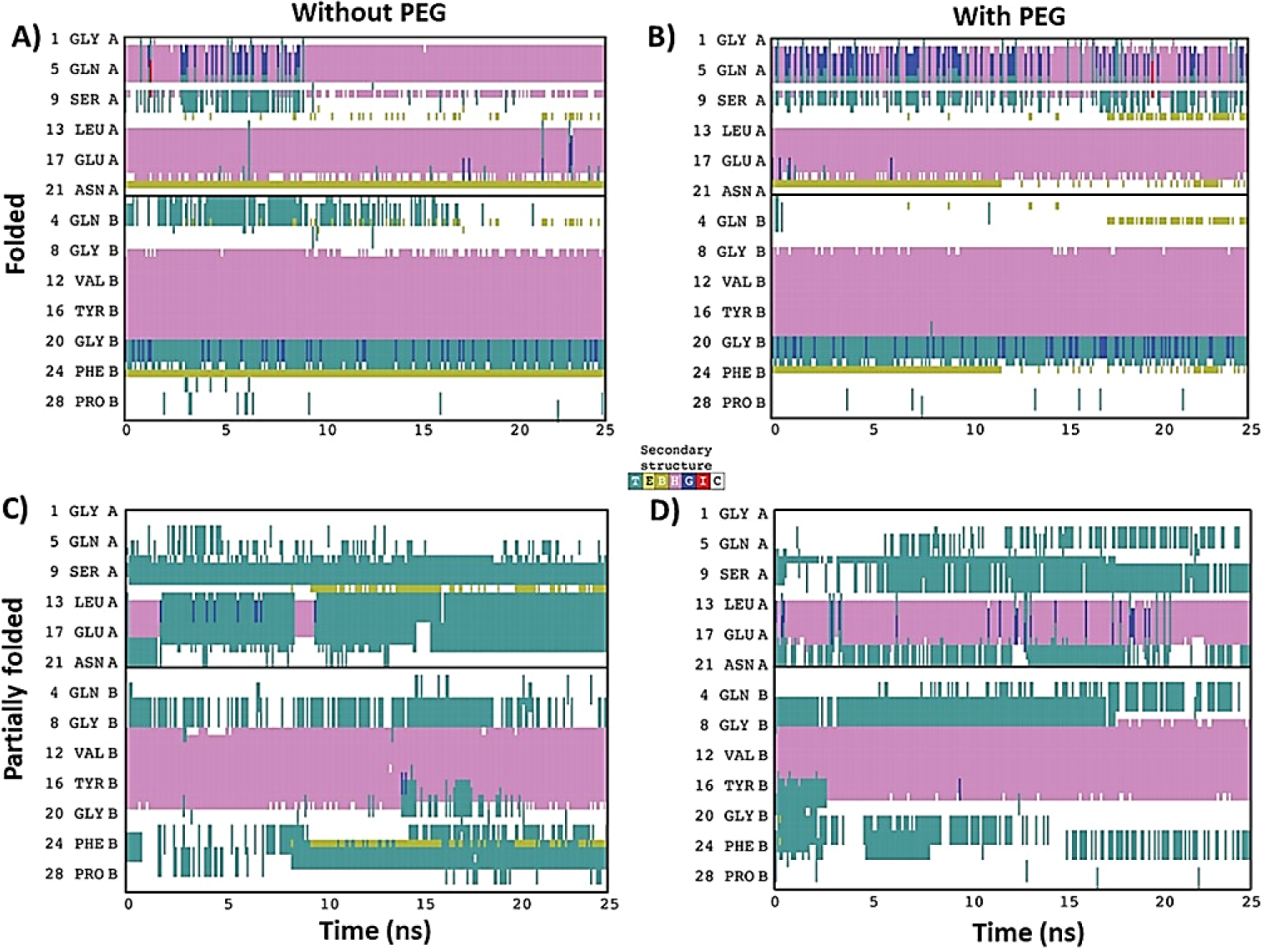
Secondary structure of insulin throughout the molecular dynamics (MD) simulation. The secondary structure of each insulin residue plotted over time for the 4 molecular dynamics (MD) simulations: (**A**) folded insulin without PEG, (**B**) folded insulin with 2 PEG molecules, (**C**) partially folded insulin without PEG, and (**D**) partially folded insulin with two PEG molecules. Residues in alpha-helices (magenta), turns (teal), beta-bridges (gold), 3–10 helices (blue), and pi-helices (red) are colored, while residues in random coils are white.

The differences between folded and partially folded insulin simulations in the absence of PEG were quite striking when looking at secondary structural elements (Figure 8). The N-terminus of chain A, which had already lacked a short α helix in the partially folded structure, appeared to become further destabilized, losing the α helix between L13 and Q17. Additionally, interchain contacts were slightly different, as evidenced by differences in isolated bridges (single-pair β-sheet hydrogen bonding). In the simulation featuring the folded insulin, C20 of chain A and F24 of chain B maintained a backbone hydrogen bond, while a second more transient interaction was observed between C11 of chain A and Q4 of chain B. None of these interactions were observed in the simulation of partially folded insulin. Instead, an interaction was observed between C11 of chain A and F24 of chain B, which happened to be on opposite ends of insulin in its folded state.

When PEG was included in the simulations (Figure 8), it resulted in secondary structure differences in both folded and partially folded insulin. The addition of PEG to properly folded insulin resulted in changes to the N-termini of chain A, as well as differences in the interdomain contacts. In the unbound simulation of folded insulin, the N-terminal helix of chain A undergoes a transient transition between α helix and 3–10 helix, before eventually settling in an α-helical state. However, in the presence of PEG, the N-terminal helix stayed in a slightly perturbed state, alternating between alpha and 3–10 helices. The pattern of isolated bridge formation also varied. Adding PEG resulted in the partial loss of the isolated bridge between C20 of chain A and F24 of chain B, while the second, more transient interaction between C11 of chain A and Q4 of chain B was increased in frequency. These results suggested that the interaction with linear PEG creates perturbations in the A chain of properly folded insulin compared with the absence of PEG, which is nearly consistent for the preference of PEG to interact with the A chain of folded insulin in the docking studies. It should be further investigated if such a perturbation could lead to the detachment of the N-termini of the A and B chains from the core that was previously reported.^52^

Adding PEG to partially folded insulin primarily resulted in two changes. The simulation with PEG resulted in the maintenance of the α helix in chain A between L13 and Y19. This suggested that it may allow for stabilization of the A chain. However, the C-terminus of chain B beyond C19 became completely detached, as seen in Figure 6G, resulting in the loss of most of its secondary structure (Figure 8). Such a detachment would expose C-terminal residues on the B chain associated with fibrillation.^53^ Although we were focused on the interaction between PEG and insulin, we performed a similar simulation to look at the effect of the gold nanoparticle itself on insulin. Properly folded insulin adsorbed to the gold nanoparticle with the 2 C-terminal residues of its B chain, while partially folded insulin adsorbed to insulin with seven residues on its A chain. The secondary structure of properly folded insulin with the gold nanoparticle resembled the simulation of properly folded insulin in solution (Figure S3). The secondary structure of partially folded insulin with the gold nanoparticle resembled a hybrid of the PEG-containing and PEG-free simulations (Figure S3). Specifically, the interaction between chain A and the nanoparticle led to much more helical structures than in solution, but at the same time, the C-terminus of the B chain retains a secondary structure like the simulation that did not contain PEG. These results suggest that the gold nanoparticle may have less of a destabilizing effect than PEG.

## SDS-PAGE Analysis

### Evaluation of Insulin Oligomerization and Proteinase K Digestion Profile

**Figure 9:**
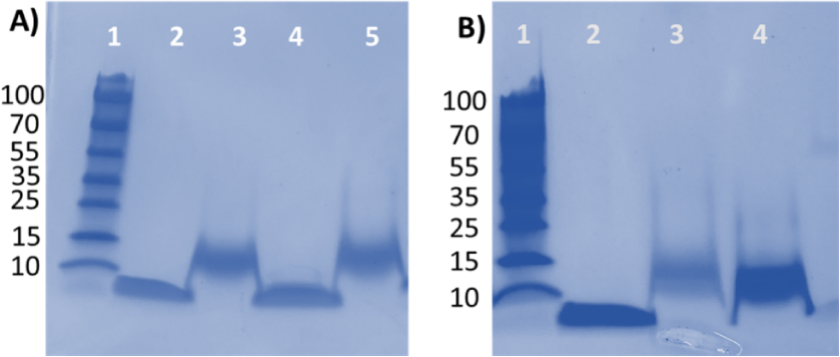
(**A**) SDS-PAGE analysis of monomeric and oligomeric states of insulin: representative gel out of *n* = 3 (**1**) ladder, (**2**) insulin at 0 h, (**3**) insulin after 3 h, (**4**) insulin at 0 h incubated with GNPs-PEG_10000_, and (**5**) insulin at 3 h incubated with GNPs-PEG_10000_. (**B**) Comparison of proteinase K digestion profiles of insulin fibrils in the absence and presence of GNPs-PEG_10000_: (**1**) ladder, (**2**) insulin monomer only, (**3**) insulin fibril with proteinase K, and (**4**) insulin fibril incubated with GNPs-PEG_10000_ with proteinase K.

The effect of GNPs-PEG_10000_ on insulin fibril formation was further assessed by 15% SDS PAGE (Figure 9A). Insulin samples incubated in the presence and absence of GNPs-PEG_10000_ for 3 h at 50 °C with continuous shaking at 1200 rpm showed the presence of higher molecular weight oligomers. However, samples at the 0 h time point mostly existed in the form of monomers. Previous studies have shown a direct correlation between proteinase K digestion and toxicity of oligomers formed during the fibrillogenisis pathway.^54^ Proteinase K digestion profile of insulin fibrils (Figure 9B) in the presence of GNPs-PEG_10000_ showed an intense band indicating protease-resistant core (lane 4, Figure 9B). However, proteinase K experiments with insulin fibrils in the absence of GNPs-PEG_10000_ suggested that they were effectively digested, as evident by the faint band (lane 3, Figure 9B). Overall, these findings suggest that there may be some variability in the structural features of insulin fibrils, including higher stability of cross-β-sheet structures generated in the presence of GNPs-PEG_10000_. However, lower molecular weight bands corresponding to the digested monomer species of insulin was not observed in the SDS-PAGE analysis under both the conditions indicating that GNPs-PEG does not protect the monomer structure from digestion.

### Assessment of Insulin Fibrillation Using Fluorescence Microscopy

**Figure 10:**
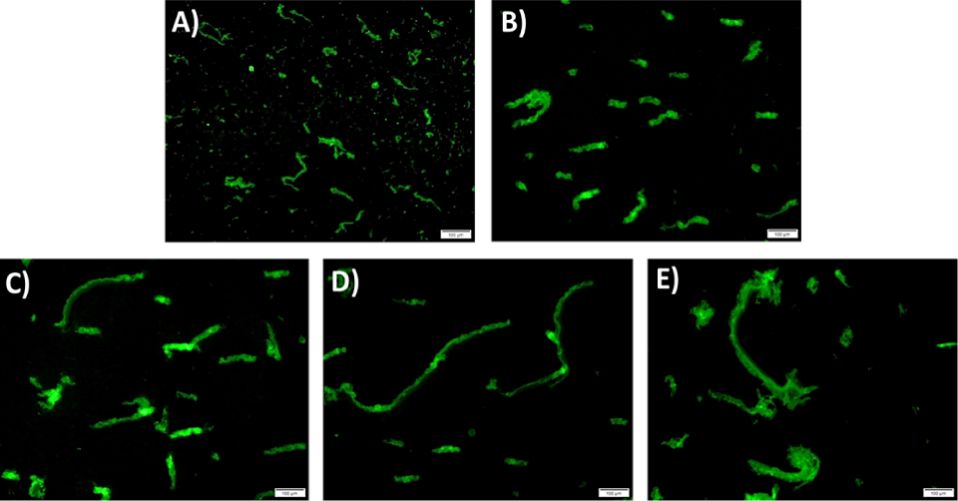
Representative fluorescence microscopy images of insulin fibrils (scale bar: 100 μm) (**A**) without GNPs-PEG and with (**B**) GNPs-PEG_200_, (**C**) GNPs-PEG_1500_, (**D**) GNPs-PEG_6000_, and (**E**) GNPs-PEG_10000_.

The overall influence of GNPs-PEG-mediated insulin fibril nucleation was further analyzed by fluorescence microscopy studies. Based on ThT studies, 3 h fibrillation at 50 °C was chosen for comparing fibril morphology in the presence and absence of GNPs-PEG. As shown in Figure 10, high molecular weight GNPs-PEGs resulted in a conglomerate of fibrils, whereas low molecular weight GNPs-PEG led to the formation of shorter fibrils. However, insulin in the absence of nanoparticles formed small oligomers and shorter fibrils.

### Insulin Fibrillation and Morphology Using Atomic Force Microscopy (AFM)

**Figure 11:**
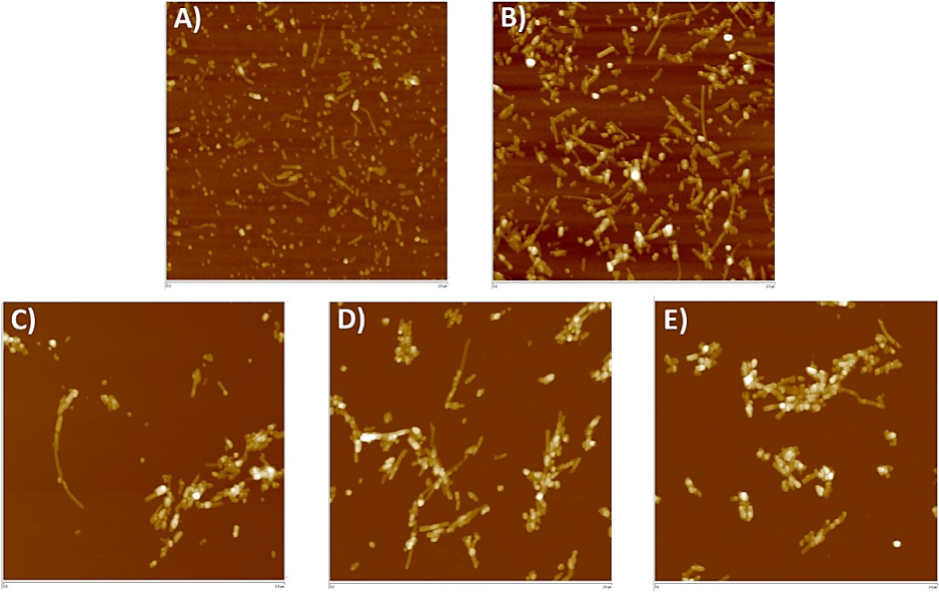
Representative AFM scans (scale bar: 2.0 μm) showing the morphology of insulin fibrils and oligomers in the (**A**) absence of GNPs-PEG and presence of (**B**) GNPs-PEG200, (**C**) GNPs-PEG1500, (**D**) GNPs-PEG6000, and (**E**) GNPs-PEG10000. Each experimental condition was repeated thrice.

Morphological characteristics of the amyloid fibrils formed in the presence and absence of GNPs-PEG were analyzed by AFM (Figure 11). AFM images were taken after incubation of the insulin sample for 3 h at 50 °C and pH 3.0. In the absence of GNPs-PEG, small globular aggregates and shorter fibrils were observed (Figure 11A). Images acquired in the presence of GNPs-PEG revealed that insulin monomers and oligomers interacted more to form longer fibrils. Moreover, as the PEG molecular weight increased (1500, 6000, and 10 000 Da), nanoparticles largely colocalized with the amyloid fibrils. Consequently, the interaction turned from individual to a conglomerate of multiple fibrils. Furthermore, in the presence of higher molecular weight GNPs-PEG, oligomers clustered within the fibril network.

### Insulin Adsorption on GNPs-PEG Surface and Assessment of Fibril Toxicity

Nanoparticles have been shown to enhance the rate of amyloid protein fibrillation by increasing the local protein concentration on their surface. Adsorption of insulin in the presence of low and high molecular weight GNPs-PEG was compared and quantified by the BCA assay. It was observed that the amount of insulin adsorbed on GNPs-PEG_200_ was relatively lower than GNPs-PEG_10000_ (Figure S4A). One-way ANOVA was performed to compare the relative amounts of insulin adsorbed on the surface of GNPs-PEG, and statistically significant differences were observed for the comparisons (*p < 0.0001). These results further suggested that not only the surface charge but also the PEG chain length and its conformation on the surface of the nanoparticle affects the adsorption rates. Insulin has an isoelectric point (pI) of 5.6; therefore, at pH 3.0, it carries a net positive charge. Adsorption is most likely facilitated by the electrostatic interactions between insulin molecules and GNPs-PEG. Increasing amount of adsorbed insulin on GNPs-PEG_10000_ compared to its higher molecular weight counterpart is likely the larger PEG chains tend to adopt “*Mushroom*”-like conformation^40^ and have more available surface area for greater protein adsorption. In contrast, the use of shorter PEG chain length results in low amounts of adsorbed insulin due to a relatively dense “brush”-like PEG layer on the surface of GNPs.

To investigate the role of PEG dynamics in insulin-amyloid-mediated cytotoxicity, cell viability experiments (MTT assays) were performed using SH-SY5Y neuroblastoma cells in the presence of GNPs-PEG. Coincubation of insulin and higher molecular weight GNPs-PEG resulted in lower toxicity, as illustrated in Figure S4B. Significant cell viability was observed in the presence of GNPs-PEG_10000_ in SH-SY5Y cells rather than insulin fibrils by itself. Toxicity levels were similar for insulin fibril alone samples and insulin fibrils generated in the presence of GNPs-PEG_200_. Initially, cell viability studies were performed with GNPs-PEG in the absence of insulin, which did not show any toxicity. To further validate data obtained from cell viability experiments, lactate dehydrogenase (LDH) release assay was conducted in the presence of oligomers and insulin fibrils cotreated with GNPs-PEG_10000_. LDH, a cytosolic enzyme is released due to mitochondrial dysfunction and loss of membrane integrity. GNPs-PEG_10000_ treatment resulted in a 22% reduction in LDH release (Figure S4C). It is interesting to point out that the above findings from the MTT and LDH assays suggest that acceleration of fibrillation by GNPs-PEG reduces the toxic oligomeric insulin intermediates and hence result in enhanced cell viability. One-way ANOVA followed by the Dunnett posthoc test was performed for multiple comparisons in both the assays, and statistically significant differences were noted (***p < 0.001 for LDH and ****p < 0.0001 for MTT).

## Conclusions

In this work, to gain a comprehensive understanding of self-assembly mechanisms of amyloid proteins in biological systems that are inherently dynamic, distinct PEG topologies on nanoparticle surfaces were engineered. Our results provide promising molecular and mechanistic descriptions of how dynamic PEG conformations on nanoparticle surfaces affect the adsorption and nucleation events during the self-assembly of amyloidogenic insulin. The results indicated that fibrillogenisis of insulin under moderately extreme conditions (50 °C and pH 3.0) in the absence and presence of GNPs-PEG proceeds via the formation of toxic oligomeric intermediates. The ThT kinetic experiments exhibited characteristics of a typical amyloid protein nucleation pathway with an initial lag phase, rapid elongation phase, and finally, a saturation phase. GNPs-PEG accelerated the insulin fibrillation process by reducing the lag phase. It was observed that the presence of these nanostructures promoted the collapse of α-helical structure as shown by CD studies. Notably, reduction of Nile red fluorescence indicated that the fibrillation of insulin in the presence of GNPs-PEG was accompanied by the decrease in the extent of exposed hydrophobic regions of the protein. MD simulation studies revealed that docking of PEG molecule to partially folded insulin resulted in the loss of most of the secondary structure elements of the B chain of insulin and, therefore, plays a major role in facilitating its fibrillation. Morphological characteristics evaluated by AFM and fluorescence microscopy showed the formation of longer and agglomerates of multiple fibrils in the presence of higher molecular weight GNPs-PEG. Furthermore, the differences in the acceleration effect of GNPs-PEG on insulin fibrillation had a direct correlation with the SPR binding data. Analysis of cell viability and LDH release assays showed reduced cellular toxicity in the presence of these nanoparticles. Overall, our results highlight the importance of the surface charge and dynamic PEG nanostructures. Both of these factors contribute to accelerated fibrillation kinetics of insulin protein by promoting conformational changes and adsorption of insulin monomers on the surface of nanoparticles. Furthermore, modulation of aggregation kinetics by GNPs-PEG through the accelerated formation of fibrillar structures minimizes toxic oligomeric intermediates and therefore opens a potential new strategy for examining their role against oligomer-induced cytotoxicity for other amyloid proteins. In conclusion, through the systematic engineering of hierarchical PEG structures on the nanoscale surfaces, we have shown a new approach of tuning fibrillation processes via the development of conformation regimes with maximum changes in the adsorption and nucleation profiles, which can be applied for other amyloid proteins.

## Experimental Section

### Materials and Methods

Phosphate buffer saline (PBS), hydrochloric acid (HCl), sodium hydroxide (NaOH), sodium chloride (NaCl), polyethylene glycol (PEG) of various sizes (200, 1500, 6000, and 10 000 Da) and isopropanol were obtained from Fisher Scientific and used without further modification. Hydrogen tetrachloroaurate (III) hydrate (HAuCl_4_.3H_2_O) was purchased from STREM chemicals, Inc. Insulin (human, recombinant), zinc-free was obtained from VWR. Glycine, thioflavin T, Congo red, Nile red, and 2, 5-diphenyltetrazolium bromide (MTT) were obtained from Sigma-Aldrich and used as received. SH-SY5Y neuronal cells were obtained from ATCC. Dulbecco’s modified eagle (DMEM) medium for cell culture studies was obtained from Corning.

Size as well as zeta potential of PEG-GNPs were determined using Malvern’s dynamic light scattering (DLS) Zetasizer-ZS90 equipped with a laser beam of He–Ne. Jasco J-815 CD spectrometer was used to perform circular dichroism (CD) experiments. Surface plasmon resonance (SPR) experiments were conducted on Reichert’s SR7500 DC SPR system with a dual channel. UV–vis measurements and fluorescence assays were performed on a Spectramax M5 plate reader. Fluorescence microscopy images were acquired using an Olympus IX73 fluorescence microscope. AFM study was performed on a Bruker Dimension Icon AFM.

### Synthesis of PEG-Coated Gold Nanoparticles (GNPs-PEG)

For each GNP solution, 6.0 g of PEG was dissolved in 45 mL of deionized water. Then, 0.75 mL of 1.0% NaOH was added to each PEG solution (slightly alkaline conditions are necessary for the formation of GNPs). Mixtures were heated and maintained at 50 °C with constant stirring for ∼10 min before 5 mL of 10 mM gold(III) chlorate (HAuCl_4_.3H_2_O, obtained from Sigma-Aldrich) was added and prepared as a stock solution (10 mM) in deionized water prior to synthesis. The mixture was slowly heated to 90 °C for 15 min until the solution changed to the distinct “ruby red” characteristic of colloidal GNPs (SI, Figure S1B). UV–vis spectra for each GNPs-PEG solution were taken on the Spectramax M5 plate reader to check the SPR wavelength of nanoparticles. This was done by placing 150 μL aliquots into a 94-well clear plate and reading absorbance values from 400 to 700 nm. The λ_max_ values between 515 and 540 nm range were indicative of stable nanoparticles, while GNPs with λ_max_ > 540 nm were deemed unstable and discarded.

### Formation of Insulin Fibrils and Thioflavin T Fluorescence Assay

Immediately prior to incubation, insulin was dissolved in 1 mM glycine-HCl buffer (pH 3.0) at a stock concentration of ∼30 μM. Precise concentrations were determined by using the absorbance at 280 nm and an extinction coefficient (ε_280_) of 5734 M^–1^ cm^–1^. The insulin stocks (1.5 mL) were combined in evaporation-tight Eppendorf tubes with the corresponding GNPs-PEG solutions and/or DI water (only DI water for controls) to yield working concentrations of 20 μM insulin. Samples were incubated with GNPs-PEG_200_, GNPs-PEG_1500_, GNPs-PEG_6000_, and GNPs-PEG_10000_ at a concentration of 0.20 nM. Tubes were sealed and placed on a thermal mixer at 50 °C and 1200 rpm throughout incubation. 100 μL aliquots were withdrawn from each sample at 15-min intervals to check the progress of fibrillation, with the first sample taken at the beginning of incubation (*t* = 0). Thioflavin T (ThT) was used to monitor this progress based on fluorescence intensity. Each 100 μL aliquot (representing a specific time point) was immediately mixed with a concentrated ThT stock solution (2 mM) to yield a 2:1 molar ratio of ThT: insulin (40 μM:20 μM). ThT-insulin mixtures were rested for 15 min at 37 °C to allow for complete binding of ThT to insulin aggregates. The samples were then transferred to a black, 384-well plate, and fluorescence intensity was recorded on the Spectramax M5 plate reader. Fluorescence was read at an excitation wavelength of 440 nm and an emission wavelength of 485 nm for all samples. These fluorescence values were fitted with a sigmoidal equation via a “least-squares” fitting method in Excel, and plots were constructed to delineate the phases of fibril growth for each sample. The generic sigmoidal equation used for obtaining the kinetic parameters is given below.
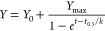


where *Y* is the fluorescence intensity at different time points, *Y*_0_ represents the intensity of the insulin monomers at time point 0 min, *Y*_max_ is the maximum fluorescence emission intensity of insulin fibrils, *t*_0.5_ represents the time required to achieve half of the maximum fluorescence intensity, and *k* is the rate constant. ThT experiments in the presence and absence of GNPs-PEG were repeated three times using different batches of nanoparticles with three replicates at each time point.

### Congo Red (CR) Binding Assay

For conducting congo red binding assay, insulin solution (20 μM) in the presence of GNPs-PEG was added to 10 μM CR in 1 mM glycine-HCl buffer (pH 3.0), and the resulting mixture was incubated for 30 min. Subsequently, UV–vis absorption spectra were recorded. Each spectrum was recorded from 400 to 650 nm. Control experiments were also conducted with an insulin solution in the absence of nanoparticles. Three independent experiments were performed, and a similar trend was observed.

### Nile Red (NR) Fluorescence Assay

For the Nile red fluorescence measurements, insulin fibril samples in the presence and absence of nanoparticles were mixed with NR, and the final concentration was kept at 10 μM. Following the addition of NR, samples were allowed to incubate briefly for 30 min. At the end of incubation, the fluorescence was read at an excitation wavelength of 530 nm, and emission spectra were recorded from 550 to 800 nm. Three independent experiments in triplicate were measured.

### Circular Dichroism (CD) Measurements

Alterations in insulin secondary structure due to GNPs-PEG were measured by collecting far-UV CD spectra at selected time points in the wavelength region between 200 and 250 nm using a quartz cuvette of 0.1 cm path length. Three replicates were run for each sample.

### Dynamic Light Scattering (DLS) and Zeta Potential Measurements

Particle size distribution of GNPs-PEG, insulin aggregation, and formation of its oligomers were determined using DLS studies at frequent time intervals during the aggregation of insulin in the presence and absence of PEG-coated nanoparticles. For all of these measurements, low-volume batch cuvettes were used. In addition, hydrodynamic diameters of the synthesized GNPs-PEG were determined at low pH and 50 °C at frequent time intervals for the assessment of their colloidal stability. In addition, zeta potential measurements of nanoparticles were also carried out to determine their surface charge. Before the zeta potential measurements, samples were diluted with DI water and carefully loaded in the zeta electrode without air bubbles. Each experiment was repeated at least three times, and the average value was used for the data analysis.

### Fluorescence Microscopy

Fibril morphology and its distribution were analyzed by acquiring fluorescence microscopy images. Briefly, insulin samples were incubated at pH 3.0 with and without GNPs-PEG for 3 h at 50 °C. Subsequently, 40 μM ThT solution was added to the mixture, and fluorescence images were taken using a fluorescence microscope. Experiments were performed three times.

### Surface Plasmon Resonance (SPR) Experiments

The interaction between GNPs-PEG and insulin was measured by SPR studies at 25 °C. Briefly, the CM5 sensor chip was activated by injecting a mixture of 40 mg/mL of EDC and 10 mg/mL of NHS. An injection of 1.0 M ethanolamine (pH 8.5) for 10 min was done for blocking the unreacted sites on the sensor chip. Subsequently, insulin protein was immobilized on the CM5 sensor chip at a concentration of 1 mg/mL around 1000 μRIU. Different GNPs-PEG with varied surface charge at a concentration of 0.2 nM were injected at a flow rate of 50 μL/min. Sensor surface was regenerated after each analyte run with an injection of 10 mM NaOH at a rate of 50 μL/min for 15 s. SPR binding assays were performed three times.

### Molecular Modeling and Molecular Dynamics Simulations

Modeling the interaction of folded insulin (PDB ID: 3I40 (PMID 20 208 155) with PEG polymers was performed using the Autodock Vina 1.1.2 software program (PMID 19 499 576) using an 84 × 64 × 60 3D grid centered on −18.189, −0.61, and −8.86 to allow global docking to both insulin chains A and B. Autodock Vina Tools 1.5.7 was used for insulin and PEG ligand preparation using the default parameters, adding total Kollman and Gasteiger charges to insulin protein. PEG1500 was drawn on ChemDraw 22.2.0 and converted to pdb online using NCI’s Online SMILES Translator and Structure File Generator tool.^55^ We evaluated about ten predicted conformations of the PEG-insulin complex, and the most stable conformation based on the lowest binding energy and the high number of hydrogen bonds was shown. To rigorously interrogate this proposed interaction and to expand it to misfolded insulin, we subsequently modeled. Modeling the interaction of folded insulin (PDB ID: 2G4M) and insulin under amyloidogenic conditions (PDB ID 1SF1) with PEG was performed 1000 times using Autodock Vina 1.1.2 with more sampling (exhaustiveness of 16). To reduce the computational time of both docking and MD simulations, PEG molecules were prepared as above, and the results from each set of 1000 simulations were consolidated. Given the lack of consensus, two nonoverlapping conformations were selected for molecular dynamics simulation.

Molecular dynamics was performed for properly folded and partially unfolded insulin, with and without PEG900, using GROMACS^56^ with the charmm36-jul2022 force field (DOI: 10.1016/j.softx.2015.06.001). The system was built using CHARMM-GUI,^57,58^ including the solvation and neutralization with two potassium atoms into a 62 Å x 62 Å x 62 Å cube (PMID 18 351 591 and 26 631 602). The steepest decent minimization was performed, and the system was equilibrated at 303.15 K with a 120 ps NVT simulation. Equilibrated systems were subjected to a 25 ns production NPT simulation. The nanoparticle was built using the Nanomaterial Modeler on a CHARMM-GUI. A sphere of gold with radius 18 Å was generated, and then, systems with properly folded and partially misfolded insulin were created by translating the insulin molecules completely outside the nanoparticle.^59^ Finally, CHARMM-GUI was used to solvate that system, and 25 ns simulations were performed using GROMACS.

### Insulin Adsorption Experiments

BCA assay kit was used for determining the amount of insulin adsorbed on the surface of GNPs-PEG. Briefly, insulin at a concentration of 2 mg/mL was dissolved in 1 mM glycine-HCl buffer (pH 3.0) and incubated with GNPs-PEG (0.2 nM) at 50 °C with continuous agitation. Reaction mixture was centrifuged at 9000 g for 10 min to remove the unbound protein, and the supernatant was carefully removed. The concentration of the protein adsorbed on the surface of the nanoparticles was estimated by making a calibration curve using bovine serum albumin. Adsorption experiments were repeated in triplicates, and values of insulin concentration are represented as mean ± SD.

### SDS-PAGE Analysis

Insulin samples (20 μM) with and without GNPs-PEG_10000_ (0.2 nM) were incubated at 50 °C in glycine-HCl buffer (pH 3.0). Samples were collected at 0 and 3 h and analyzed by 15% SDS-PAGE gel electrophoresis. Three independent gels were run separately for checking experimental variation.

### Proteinase K Digestion Assay

Insulin (20 μM) in 1.0 mM glycine-HCl buffer (pH 3.0) in the absence and presence of GNPs-PEG_10000_ was incubated for 3 h at 50 °C. Next, samples were subjected to proteinase K digestion (2.5 μg/mL) by incubating for 30 min at 37 °C. Reaction was stopped by adding 2X SDS sample buffer, followed by heating at 100 °C for 10 min. Samples were then assessed on 15% SDS-PAGE gels. Proteinase K digestion protocol was repeated three times with independent samples, followed by SDS-PAGE analysis.

### Cytotoxicity (MTT) Assay

Cytotoxicity of insulin aggregates and fibrils in the presence and absence of GNPs-PEG was determined in the SH-SY5Y cell line. Cells were seeded in 96-well plates at a density of 2,500 cells per well. After 24 h, cells were treated with insulin fibrils generated in the presence and absence of nanoparticles and incubated overnight at 37 °C in a humidified incubator with 5% CO_2_. Subsequently, each well was washed twice with PBS, and then, 50 μL of a 5 mM MTT solution was added. Finally, after 4–6 h of incubation, the resulting formazan crystals were dissolved in 75 μL of acidic isopropanol, and the absorbance was recorded at 570 nm using a microplate reader. Three independent experiments were done in triplicates. % cell viability is illustrated as mean ± SD from the average of 3 wells. Statistical significance was determined using one-way Anova, followed by the Dunnett posthoc test.

### Lactate Dehydrogenase Assay

Cytotoxicity of insulin aggregates and fibrils in the presence and absence of GNPs-PEG was also determined in SH-SY5Y cell line by LDH release assay using LDH cytotoxicity assay kit from Thermos Fisher Scientific according to manufacturer’s recommended protocol. Experiments were repeated thrice, and % LDH represents mean ± SD from the average of 3 wells. One-way Anova was used for the determination of statistical significance.

### Atomic Force Microscopy (AFM)

Morphological alterations during insulin fibrillation in the presence and absence of GNPs-PEG were analyzed using AFM microscopy. Insulin sample (20 μM) was further diluted to 2.0 μM. Samples for imaging were prepared by pipetting 20 μL of each sample solution onto freshly cleaved mica substrates and letting them incubate for 5 min. The mica substrate was subsequently washed with 18 MΩ deionized water and dried using ultra high purity (UHP) dry nitrogen gas. The scans were conducted with a Bruker Dimension Icon AFM instrument in tapping mode. HiRes150 Si tips (Budget Sensors) with a nominal tip diameter (1 nm) and force constant (5 N/m) were used for imaging. The scans were acquired at 512 × 512 pixels resolution with a scan rate of 1.0 Hz. Subsequently, the substrate was washed with deionized water and air dried. AFM imaging was acquired under tapping mode using a silicon AFM cantilever. AFM experiments were performed three times, and the acquired images had consistent trends in all three replicates. For each sample, 5–6 different areas were randomly selected for scanning.

### Statistical Data Analysis

All the data reported in this study were obtained from three replicates. For ThT experiment, values are expressed as mean ± SD where SD was obtained from the average of triplicates. For DLS measurements, values are given as mean ± SD (*n* = 3 from independent experiments). To ensure rigor and reproducibility, AFM, fluorescence, SDS-PAGE, proteinase K, and TEM experiments were repeated thrice, and representative data have been presented. MTT, LDH, and adsorption experiments were repeated three times, and presented data are shown as mean ± SD from the average of 3 wells. Statistical data analysis for cell viability assay, LDH, and adsorption experiments was performed using Graph Pad prism software. One-way Anova, followed by the Dunnett posthoc test was conducted for multiple comparisons with 95% confidence intervals: *p < 0.05, ***p < 0.001, and ****p < 0.00001 indicate statistically significant differences.

## Supporting Information Available

Materials and instrumentations; detailed experimental section; synthesis and characterizations of GNPs-PEG; stability study of GNPs-PEG; insulin adsorption on GNPs-PEG surface and assessment of fibril toxicity (PDF)
