# Supplementary material for: Mechanistic Insights Behind the Self-Assembly of Human Insulin under the Influence of Surface-Engineered Gold Nanoparticles
Source: ACS Chem Neurosci. 2024 May 10;15(11):2359–71. doi: 10.1021/acschemneuro.4c00226 (PMC11157486; doi:10.1021/acschemneuro.4c00226)
Supplement: Supplementary file 1 — cn4c00226_si_001.pdf [file cn4c00226_si_001.pdf]

"Supporting Information"

Mechanistic Insights Behind the Self-Assembly of Human Insulin Under the Influence of Surface-Engineered Gold Nanoparticles

*Zachary Flint,[1] Haylee Grannemann,[1] Kristos Baffour,[1] Neelima Koti,[1] Emma Taylor,[1] Ethan Grier,[1] Carissa Sutton,[1] David Johnson,[2] Prasad Dandawate,[3] Rishi Patel,[4] Santimukul Santra,[1] and Tuhina Banerjee[1, *]*

[1]Department of Chemistry and Biochemistry, Missouri State University, 901 S. National Avenue, Springfield, MO 65897, United States of America

[2]Molecular Graphics and Modeling Laboratory, University of Kansas, 2034 Becker Drive, Lawrence, KS 66018, United States of America

[3]Department of Cancer Biology, The University of Kansas Medical Center, Kansas City, KS 66160, United States of America

[4]Jordan Valley Innovation Center, Missouri State University, 542 N. Boonville Avenue, Springfield, MO 65806, United States of America

*Corresponding author: Tuhina Banerjee, Email: tbanerjee@missouristate.edu

Syntheses and characterization of GNPs-PEG

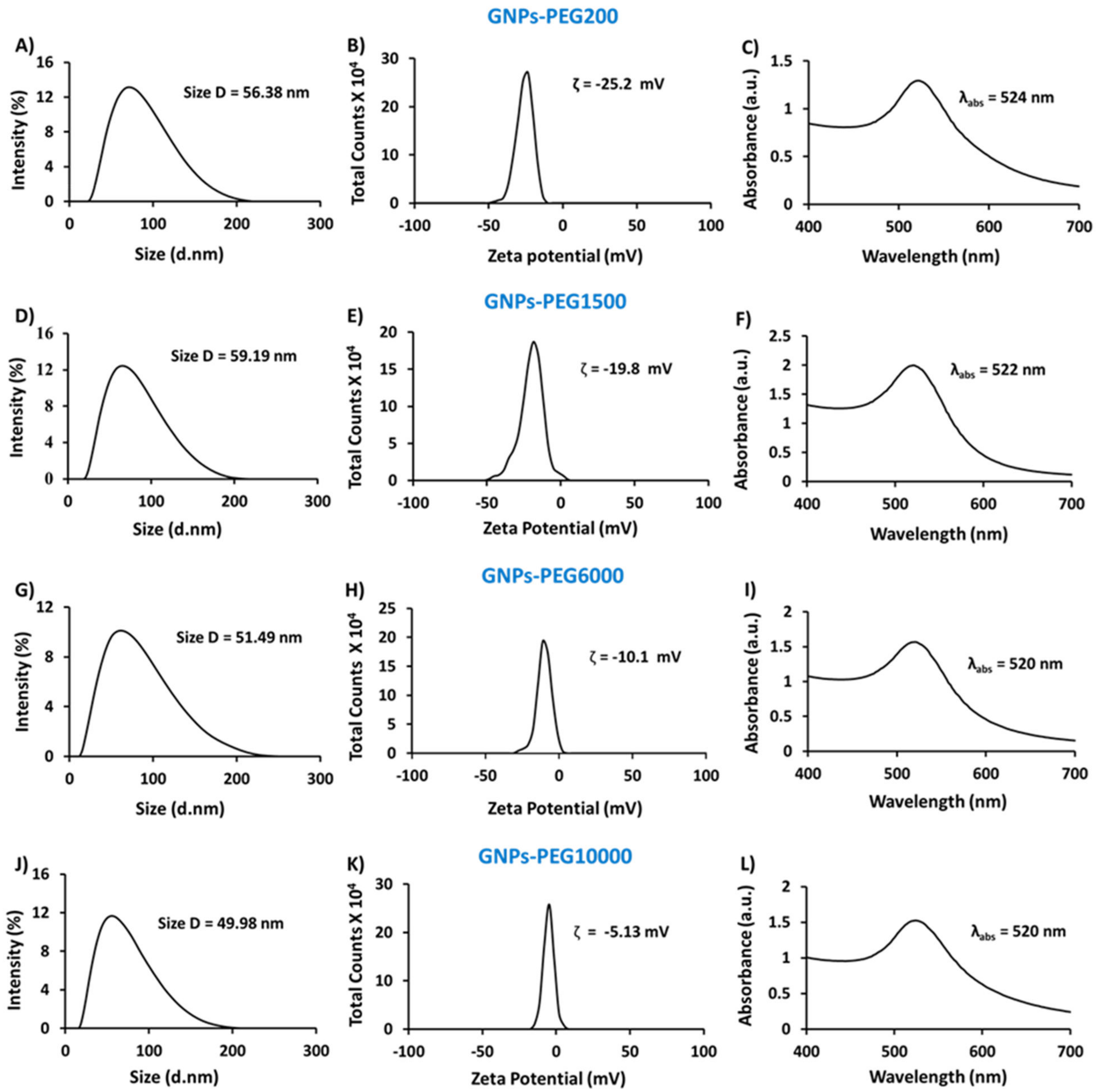

Figure S1: Characterizations of GNPs-PEG coated with different molecular weights of PEG using dynamic light scattering and UV/Vis spectrometry.

Nanoparticles (GNPs-PEG)	Size (nm)	Zeta potential (mV)	Absorption Maximum (nm)
GNPs-PEG$_{200}$	56 ± 4	-25.2 ± 1.5	522 ± 1
GNPs-PEG$_{1500}$	59 ± 1	-19.8 ± 2.3	524 ± 2
GNPs-PEG$_{6,000}$	51 ± 2	-10.1 ± 2.1	520± 3
GNPs-PEG$_{10,000}$	50 ± 4	-5.13 ± 1.5	520 ± 1

Table S1: Hydrodynamic diameter, zeta potential, and absorption maximum (surface plasmon resonance) of various GNPs-PEG.

Transmission Electron Microscopy and characterization of GNPs-PEG

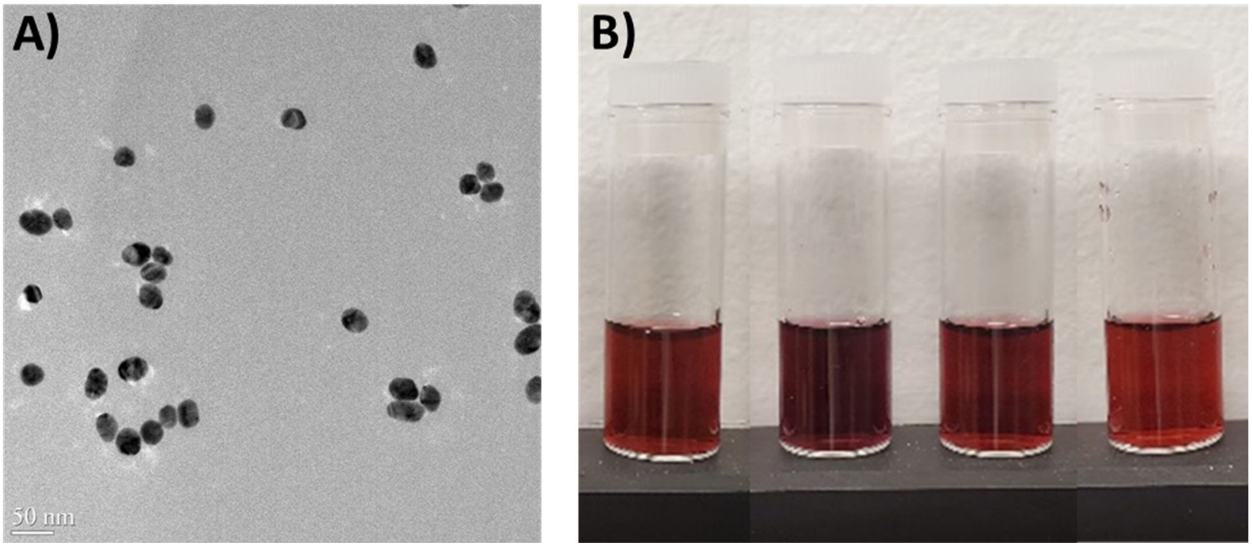

Figure S2. A) Representative TEM image of GNPs-PEG$_{200}$ (Scale bar: 50 nm), showing spherical morphology with gold core size of approx. 20 nm. **B)** Color comparison of different chain lengths of PEG (from left to right: PEG 200, PEG 1500, PEG 6000 and PEG 10000)-coated GNPs.

Nanoparticles	Size (nm) @ pH 7.4	Size (nm) @ pH 3.0
GNPs-PEG$_{200}$	53 ± 3	50 ± 1
GNPs-PEG$_{1500}$	55 ± 2	53 ± 2
GNPs-PEG$_{6000}$	49 ± 1	51 ± 3
GNPs-PEG$_{10000}$	53 ± 2	52 ± 1

Table S2: Hydrodynamic diameters of PEG-GNPs at different pH after 60 days.

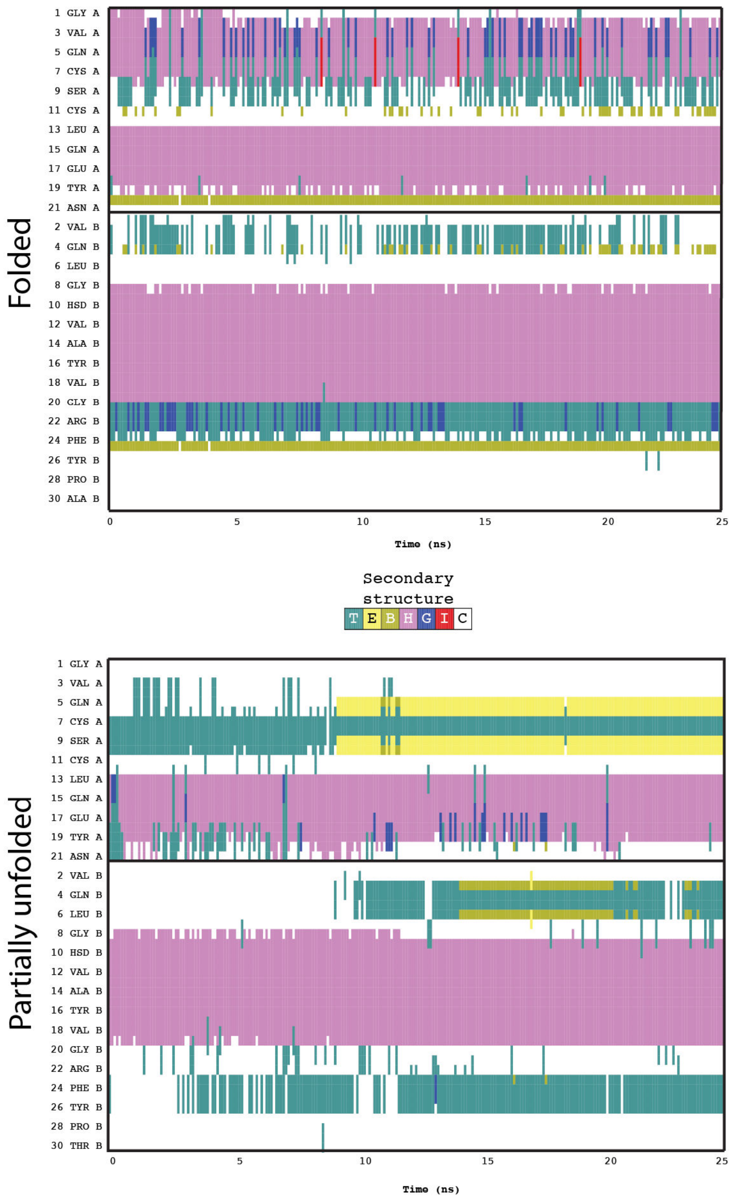

Figure S3: Secondary structure of insulin throughout the molecular dynamics (MD) simulation with the gold nanoparticle. The secondary structure of each insulin residue, plotted over time for the 2 molecular dynamics (MD) simulations: **(top)** folded insulin with a gold nanoparticle, **(bottom)** misfolded insulin with a gold nanoparticle. Residues in alpha-helices (magenta), turns (teal), beta-bridges (gold), 3-10 helices (blue), and pi-helices (red) and are colored, while residues in random coils are white.

Insulin adsorption on GNPs-PEG surface and assessment of fibril toxicity

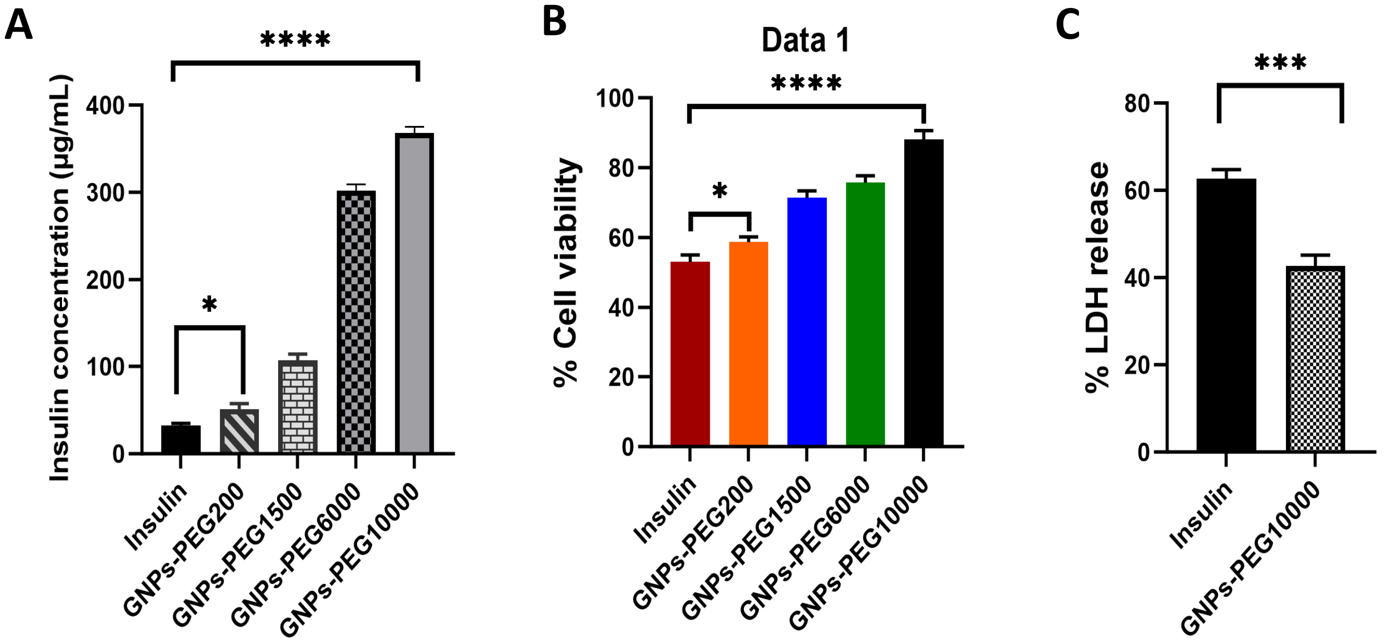

Figure S4: A) Adsorption of insulin on the surface of GNPs-PEG, and B) Cytotoxicity of insulin fibrils using SH-SY5Y cells in the presence and absence of GNPs-PEG. C) LDH release assay in SH-SY5Y cells using insulin fibrils in the presence of GNPs-PEG. In all these experiments, means ± SD were calculated from the average of 3 wells. Statistical data analysis was performed using one-way Anova, followed by Dunnett's poc-hoc test for multiple comparison; *P<0.05, ***P<0.001 and ****P<0.0001 indicates statistically significant differences.
